# A microfluidic biochip for locally confined stimulation of cells within an epithelial monolayer†
†Electronic supplementary information (ESI) available. See DOI: 10.1039/c7ra11943g


**DOI:** 10.1039/c7ra11943g

**Published:** 2018-02-19

**Authors:** Roland Thuenauer, Simon Nicklaus, Marco Frensch, Kevin Troendle, Josef Madl, Winfried Römer

**Affiliations:** a Faculty of Biology, Albert-Ludwigs-University Freiburg, Schänzlestraße 1, 79104 Freiburg, Germany. Email: roland.thuenauer@gmail.com; Email: winfried.roemer@bioss.uni-freiburg.de; b BIOSS – Centre for Biological Signalling Studies, Albert-Ludwigs-University Freiburg, Schänzlestraβe 18, 79104 Freiburg, Germany; c Freiburg Center for Interactive Materials and Bioinspired Technology (FIT), Albert-Ludwigs-University Freiburg, Georges-Köhler-Allee 105, 79110 Freiburg, Germany; d International Max Planck Research School for Molecular and Cellular Biology, Max Planck Institute of Immunobiology and Epigenetics, Stübeweg 51, 79108 Freiburg, Germany

## Abstract

A key factor determining the fate of individual cells within an epithelium is the unique microenvironment that surrounds each cell. It regulates location-dependent differentiation into specific cellular sub-types, but, on the other hand, a disturbed microenvironment can promote malignant transformation of epithelial cells leading to cancer formation. Here, we present a tool based on a microfluidic biochip that enables novel research approaches by providing a means to control the basolateral microenvironment of a confined number of neighbouring cells within an epithelial monolayer. Through isolated single pores in a thin membrane carrying the epithelial cell layer only cells above the pores are stimulated by solutes. The very thin design of the biochip (<75 μm) enabled us to apply a high-resolution inverted confocal fluorescence microscope to show by live cell imaging that such a manipulation of the microenvironment remained locally restricted to cells located above the pores. In addition, the biochip allows access for the force probe of an atomic force microscope (AFM) from the apical side to determine the topography and mechanical properties of individual cells, which we demonstrated by combined AFM and fluorescence microscopy imaging experiments. Taken together, the presented microfluidic biochip is a powerful tool that will enable studying the initial steps of malignant transformation of epithelial cells by directly manipulating their microenvironment and by real-time monitoring of affected cells with fluorescence microscopy and AFM.

## Abstract 2

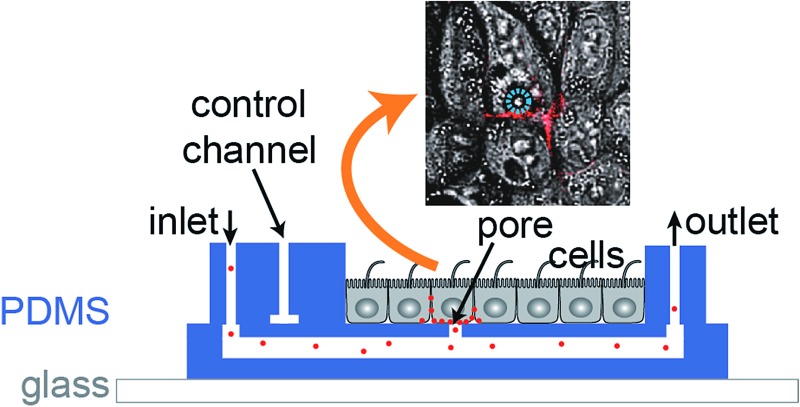
We developed a microfluidic biochip that enables one to locally change the basolateral microenvironment of epithelial cells within a polarised monolayer.

## Introduction

Epithelial cell layers constitute a basic architectural principle of multicellular organisms by allowing maintaining compartments with defined compositions. Most luminal surfaces of the human body are lined by epithelia, which control the permeation and secretion of solutes in and out of organs.^1^,^2^

Key to maintaining a functional epithelium is the microenvironment that surrounds individual epithelial cells and determines their fate. The microenvironment is defined by soluble molecules, which are sensed by cellular receptors, and cell junctions. The latter enable epithelial cells to recognise and anchor to the extracellular matrix, such as focal adhesions, and to link to neighbouring cells, such as tight junctions and adherens junctions. Cell junctions are highly dynamic structures; their components are constantly turned over by an intracellular trafficking network that organises recycling, degradation and delivery of newly synthesised adhesion molecules.^3^,^4^ Through these junctions epithelial cells within a monolayer permanently receive signals ensuring their growth arrest and polarised state.^3^

An additional important aspect is that most epithelia found in the human body do not consist of homogenous cell types, but show a patterned organisation of many cellular sub-types that fulfil specific functions. One example is the organisation of the alveolar epithelium in the lung, which consists of type I cells that are responsible for gas exchange and type II cells that secrete pulmonary surfactant. Microenvironments are pivotal for the establishment of appropriately patterned epithelia, because from them individual cells derive the signals that lead to their location-dependent differentiation into the correct sub-type.

On the other hand, if for example injury or damaging environmental conditions disturb the microenvironment, or the related delicate balance between signalling and trafficking of adhesion molecules is changed by mutations, severe consequences can ensue. Then, a malignant transformation can occur through only partially known mechanisms that enables affected cells to escape their polarised and growth-arrested state in the epithelium and to start proliferating, potentially leading to cancer.^3^,^5^,^6^ The outstanding importance of epithelia for cancer development is illustrated by the fact that approximately 90% of all human cancers derive from epithelial cells.^5^

However, our knowledge about the microenvironment of epithelial cells is still incomplete,^7^,^8^ as is our understanding of the very first steps of malignant epithelial cell transformation and cancer establishment.^9^–^12^ An approach to shed light on these questions would be to extrinsically adjust and control the microenvironment of selected cells within an epithelium. Whereas the apical side of epithelial cells is freely accessible, which allows utilising existing techniques to locally apply molecules through *e.g.* microfluidic probes^13^ or microfluidic pipettes,^14^ the situation on the basolateral side, at which the cells are attached to a substrate, is more challenging. Yet, the basolateral membrane contains microenvironment-sensing cell adhesion receptors, such as integrins or cadherins, which have a pivotal role in cancer development and progression, and also many hormones and nutrients from the blood stream are reaching epithelial cells at the basolateral side *via* the underlying basement membrane and connective tissue.

Here, we present a microfluidic biochip that enables delivering molecules in a locally confined manner to the basolateral side of cells within an epithelium and hence changing their basolateral microenvironment. Microfluidic biochips are well suited to replicate the natural architecture of tissue *in vitro*^15^ and numerous biochips have been developed to reconstitute aspects of the physiological organisation of epithelia.^16^–^19^ A previously successful design is based on a porous membrane that carries the epithelial monolayer and separates two chambers or channels that allow independent access to the apical and basolateral side of the epithelium. To achieve our goal, we varied this design by using a membrane as support for the epithelial monolayer that contains only single isolated pores. Hence, only the cells that grow above a pore can be selectively stimulated with solutes from the basolateral side, thus allowing to effectively control the microenvironment of these cells. In addition, our chip is sufficiently thin to enable real-time high-resolution fluorescence imaging of individual cells with an inverted microscope. Since the mechanical properties of cells tightly correlate with their malignant potential,^20^–^22^ our chip also provides access for the force probe of an atomic force microscope (AFM) from the apical side, which can be used to visualise the topography and to mechanically characterise individual cells directly within the cell layer in parallel to fluorescence imaging.

## Experimental

### Biochip architecture

**Fig. 1:**
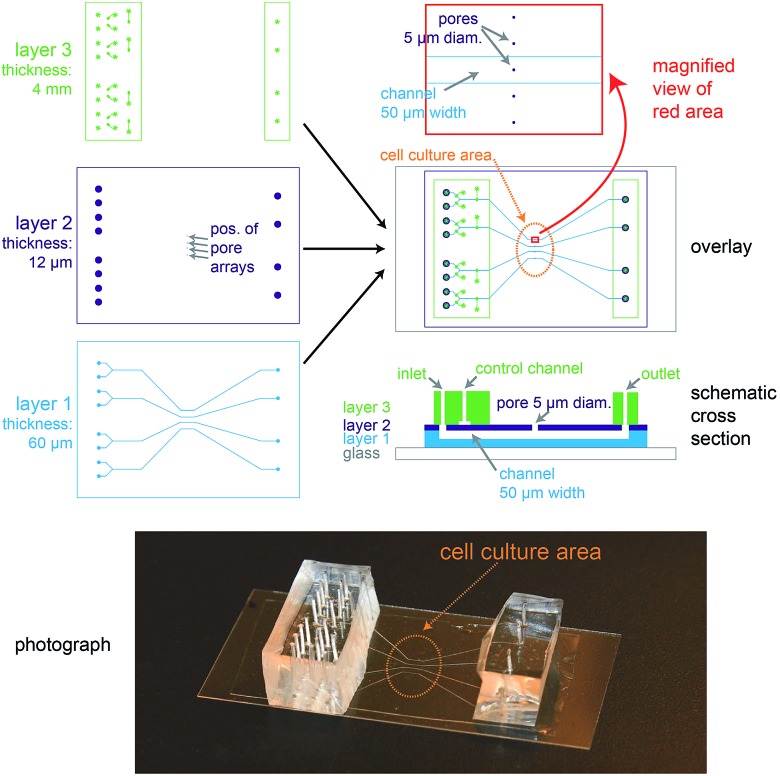
Design of the microfluidic biochip. On the top left side, the designs of the three PDMS layers from which the chip is made are shown. The light blue areas in layer 1 correspond to channels, whereas the dark blue areas in layer 2 correspond to holes or pores. The green areas in layer 3 correspond to channels, and the star-shaped structures represent the position of access ports for tubing. In the overlay image (middle right) the fibronectin-coated cell culture area is outlined by an orange oval, the magnification (top right) shows how the pores are aligned above the channels and also a schematic cross section is depicted (bottom right). On the bottom a photograph of a completely assembled chip with a supporting glass cover slip is shown.

The biochip design is schematically presented in Fig. 1. The chip consists of three layers of polydimethylsiloxane (PDMS) that are supported by a glass cover slip. Layer 1 is 60 μm thick and contains four channel systems (‘basolateral channels’) of 50 μm channel width. Layer 2 is a thin membrane (12 μm thickness) that contains pores of 5 μm diameter and access holes to the basolateral channels on the left and right. Layer 3 is 4 mm thick and consists of two rectangular patches containing access ports in which steel tubes are inserted in order to connect the channel inlets and outlets to external tubing. In addition, layer 3 includes channels that form together with the underlying thin membrane of layer 2 valves that can be used to control the flow in the basolateral channels.

In order to ease the alignment between layer 1 and layer 2 during production (see Fig. S1† for an outline of the production process), an array of five pores is available for each basolateral channel. However, the distance between these five pores is sufficiently wide so that only a single pore can end up on top of each 50 μm wide channel (Fig. 1, magnification), therefore allowing some inaccuracy during positioning layer 1 on layer 2. The orange oval in the overlay image of Fig. 1 is coated with fibronectin. This facilitates cell attachment, so that only the designated cell culture area gets covered by an epithelial cell monolayer.

For light microscopy, the chip is placed on an inverted microscope and the cells are imaged by an objective from underneath the glass cover slip. At the cell culture area, the total thickness of the chip is by design 72 μm. This ensures that objectives with high numerical aperture (NA), which typically have small working distances (<150 μm), can be used to perform high-resolution microscopy.

From the apical side the cells are freely accessible for exchanging the buffer manually *via* a pipette. In addition, the gap between the access ports on each side (layer 3) was designed sufficiently wide (>20 mm) so that the cell culture area in the centre can be directly reached by an AFM cantilever mounted on a holder.

In conclusion, the chip architecture fulfils all requirements to stimulate a confined number of cells within an epithelial monolayer from the basolateral side and to enable parallel real-time observation by fluorescence microscopy and AFM.

### Biochip manufacturing

The manufacturing process of the biochip is schematically outlined in Fig. S1.† The biochip consists of three PDMS layers that are supported by a glass cover slip (#1.5, Carl Roth). The individual PDMS layers were manufactured by casting or spinning un-crosslinked PDMS solutions (Momentive RTV 615) on masters. The masters were silicon wafers that contained suitable microstructures made of SU 8 (layers 2 and 3, 20 μm and 50 μm thickness, respectively) or ma-P 1275 (layer 1, 25 μm thickness) that were manufactured by standard photolithography by CBL GmbH (Linz, Austria). The master for layer 1 was heated to induce ma-P 1275 reflow to achieve a rounded cross section of the basolateral channels. Prior to usage, all masters were exposed to chlorotrimethylsilane (Sigma-Aldrich) vapour in order to prevent irreversible adhesion of PDMS. Layer 1 was manufactured by spin coating a master with an un-crosslinked PDMS solution (component A : B weight ratio 20 : 1) at 1200 rpm for 60 s to form an approximately 60 μm thick PDMS layer. After that, a frame of PDMS was placed on the freshly coated PDMS layer in order to ease handling of the thin layer 1 and the combined structure was pre-cured at 80 °C for 30 min. Layer 2 contains the pores and was manufactured by spin coating a master with an un-crosslinked PDMS solution diluted with cyclohexane (component A : component B : cyclohexane weight ratios 5 : 1 : 10) at 5100 rpm for 60 s to form an approximately 12 μm thick PDMS layer. Layer 2 was pre-cured at 80 °C for 10 min. Next, the pre-cured layer 1 was removed from the master with the help of the attached frame and was aligned and placed on layer 2 using a stereo microscope. The assembly was cross-linked by incubation at 90 °C for 4 h and then removed from the master and placed on a glass cover slip. Layer 3 contains the access ports for the channels and was manufactured by casting an un-crosslinked PDMS solution (component A : B ratio 10 : 1) on a master to form an approximately 4 mm thick layer. After curing for 3 h at 80 °C, the layer was removed from the master, cut to size with a scalpel, and access holes were punched with a 20 gauge needle. Layer 3 was cross-linked with layer 1 and 2 by activating the surfaces with air plasma (Zepto, Diener electronic) and then pressing them together followed by incubation for 3 h at 80 °C.

To measure the true thicknesses of layers 1 and 2, 0.1 μm fluorescent TetraSpeck beads (Thermo Fisher) were applied between each layer during production. Fig. S2A† shows a representative cross-section of a chip that was acquired with a confocal fluorescence microscope (Nikon A1R equipped with a 60× oil immersion objective, NA 1.49) and from which the layer thicknesses can be determined. In Fig. S2B,† the average measured thickness values from three independent production cycles are displayed. On average, a thickness of 62 ± 3 μm was achieved for layer 1 and 12 ± 1 μm for layer 2.

In addition – since the architecture of the on-chip valves is non-standard and is based on the 12 μm thick membrane of layer 2 – the functionality of the valves was verified by filling the channels with differently coloured fluorescent solutions (Fig. S3†).

### Cell lines, cell culture, and plasmids

All cell culture reagents were purchased from Thermo Fisher unless stated otherwise. Wild type (wt) Madin–Darby canine kidney strain II (MDCK II; kindly provided by Enrique Rodriguez-Boulan (Weill Cornell Medical College, NY, USA)) cells were cultured in Dulbecco's Modified Eagle's Medium (DMEM) supplemented with 5% fetal calf serum (FCS) at 37 °C and 5% CO_2_.

The plasmid pML-GFP encoding for the plasma membrane marker GFP tagged with a Lyn-derived myristoylation signal (ML-GFP) and G418 resistance was a gift from Christian Wunder (Curie Institute, Paris, France). MDCK cells stably expressing ML-GFP were created by transfecting wt MDCK II cells with pML-GFP using Lipofectamine 2000. Next, cells were selected with 1 mg ml^–1^ G418 for 2 weeks and clones showing homogeneous overexpression of ML-GFP were selected. Clones were cultured for 4 days on transwell filters (0.4 μm pore size, polycarbonate membrane, Corning) and the trans-epithelial electrical resistance (TEER) as well as the distribution of the polarity markers β-catenin (basolateral marker) and ZO-1 (tight junction marker) was compared to wt MDCK cells. A clone showing the best agreement of these parameters with wt MDCK cells was chosen and used for further experiments. These experiments also demonstrated that ML-GFP labelled apical and basolateral plasma membranes of MDCK cells.

For growing polarised MDCK monolayers on the biochip, the biochips were first washed with ethanol to remove soluble components from PDMS and then autoclaved for sterilisation. The cell culture area in the centre of the chip (see Fig. 1) was coated with fibronectin to ensure efficient cell attachment.^23^ To this end, bovine fibronectin (Sigma Aldrich) was diluted in phosphate buffered saline (PBS) to a concentration of 10 μg ml^–1^ and a drop of the fibronectin solution of sufficient volume to cover the cell culture area (approximately 10 mm diameter) was dispensed at the chip surface and incubated for 1 h at room temperature. After washing the chip three times with PBS and filling the channels with cell culture medium, MDCK cells were seeded on the chip by dispensing 500 μl of cell culture medium containing 5 × 10^5^ cells. The cells were allowed to attach for 1 h at 37 °C and 5% CO_2_ and then non-attached cells were washed away. A PDMS frame surrounding the cell culture area with 5 mm height was placed on the chip in order to form a well. 1 ml of cell culture medium was applied to the well and the cells were cultured for 3 days with daily exchange of the medium to form a polarised monolayer.

### Cell stimulation on the chip and real-time fluorescence microscopy

Solutions were pumped through the channels with a microfluidic flow control system (OB1, Elveflow). The valves were operated by a home-build controller^24^ (‘WAGO Controller’) using solenoid valves and a custom-written GUI in Matlab (Version 8.5, Mathworks).

For imaging live cells, the cell culture medium was replaced with live cell imaging medium (Hank's balanced salt solution (HBSS) supplemented with 20 mM HEPES, 1% FCS, 4.5 g l^–1^ glucose, 2 mM l-glutamine). Fluorescence imaging was performed with a Nikon A1R confocal microscope equipped with a 40× water immersion objective (NA 1.25, working distance 160–200 μm) and an incubator housing maintaining a constant temperature of 37 °C (Okolab). To prevent evaporation, the cell culture well on the biochip was sealed with a PDMS slab during measurement.

After flooding the flow channel with live cell imaging medium containing 1% bovine serum albumin (BSA) to reduce unspecific adsorption to PDMS, live cell imaging medium supplemented with 10 μM of the cell-binding lectin BambL from *Burkholderia ambifaria*^25^ tagged with Cy5 (BambL-Cy5) was flushed into the channel at a constant pressure of 20 mbar.

### Immunofluorescence and antibodies

Cells were fixed by incubation with 4% formaldehyde for 15 min at room temperature. After permeabilising the cells for 30 min with PBS supplemented with 1% saponin and 3% BSA, the sample was incubated with primary antibodies for 1 h followed by washing and incubation with appropriate secondary antibodies for 30 min. Nuclei were counterstained with DAPI. The following primary and secondary antibodies were used: rabbit anti-β-catenin (Abcam), rat anti-ZO-1 (Millipore), Cy3-tagged donkey anti-rabbit (Jackson ImmunoResearch), Alexa647-tagged goat anti-rat (Thermo Fisher).

### Combined atomic force and fluorescence microscopy

The measurements were performed on an AFM (JPK NanoWizard 3 BioScience AFM) that was mounted on an inverted fluorescence microscope (Nikon Eclipse Ti, Andor IXon EMCCD camera, 488 nm laser). The objective was a 40× water immersion objective (NA 1.25). The optical images were correlated with the AFM images by applying the DirectOverlay routine from JPK. In brief, images of the cantilever were recorded at predefined positions, which allowed calibration of the optical cantilever images *versus* their position within the AFM-‘field of view’. All experiments were performed on formaldehyde-fixed cells in PBS. The AFM was operated in contact mode using soft cantilevers (nominal spring constant 10 pN nm^–1^).

## Results and discussion

### Growth of polarised epithelial monolayers on the biochip

**Fig. 2:**
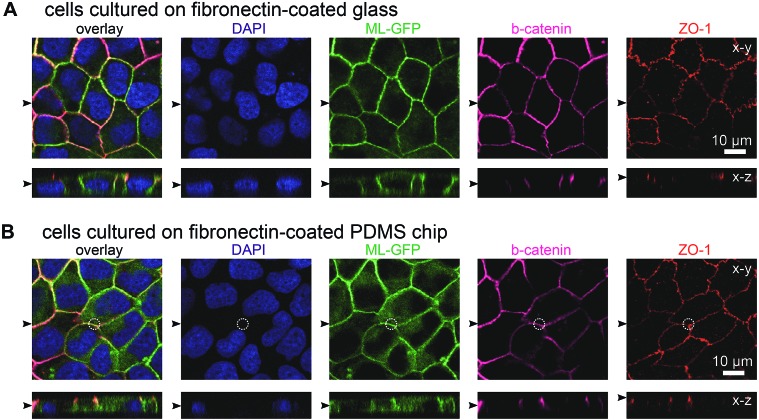
MDCK cells form polarised monolayers on the biochip. MDCK cells stably expressing ML-GFP (green) were grown for three days on a fibronectin-coated glass cover slip (A) and on a fibronectin-coated PDMS-based biochip (B). After fixation, cells were permeabilised and stained with DAPI highlighting nuclei (blue) and antibodies recognising the basolateral marker beta-catenin (magenta) and the tight junction marker ZO-1 (red). A white circle indicates the perimeter of the pore in (B). Displayed are representative *x*–*y* sections and *x*–*z* sections from the acquired confocal image stack. The black arrowheads indicate the positions of the respective image sections.

In initial experiments we verified that MDCK cells are able to form polarised monolayers on the chip (Fig. 2). Bare PDMS surfaces are too hydrophobic for proper cell attachment.^23^ Therefore, the cell culture area of the chip was coated with the extracellular matrix protein fibronectin to facilitate cell attachment.^23^ For comparison, we cultured ML-GFP-expressing MDCK cells for 3 days on fibronectin-coated glass cover slips (Fig. 2A) and on fibronectin-coated PDMS-based biochips (Fig. 2B). Stainings revealing the localisation of the basolateral marker beta-catenin^26^ and the tight junction marker ZO-1 (ref. 26) demonstrated that a well-polarised monolayer was formed on the PDMS-based biochip.

The plasma membrane marker ML-GFP allowed us to check if MDCK cells are able to grow into the pores, which occurred in some cases (not shown). Therefore we checked that pores were devoid of signal from ML-GFP before each experiment and used only samples that fulfilled this condition. Here, the fact that each chip contains four copies of basolateral channels with pores significantly increased the chance to find an appropriately overgrown pore on one chip. Importantly, if multiple cells are located over the area of the pore, intact ZO-1-labelled tight junctions still formed at this area (Fig. 2B, within the white circle outlining the pore perimeter). This is a prerequisite that no paracellular leakage to the apical side occurs when solutes are introduced *via* the pore from the basolateral side.

### Locally confined stimulation of cells within an epithelial monolayer

**Fig. 3:**
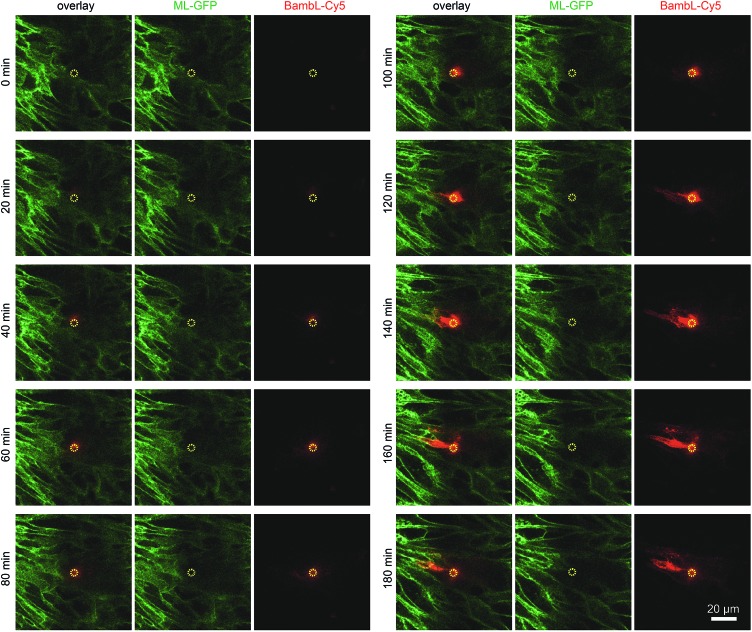
Addressing selected cells within an epithelial monolayer. MDCK cells stably expressing ML-GFP (green) were grown as polarised monolayer on the biochip. By using a confocal microscope the image plane on which the cells are attached to the chip surface was observed during introduction of medium containing 10 μM BambL-Cy5 (red) through the basolateral channel starting at *t* = 5 min. A yellow circle indicates the perimeter of the pore.

To demonstrate that cells located above the pore can be selectively stimulated, we used the fucose-specific lectin BambL conjugated to the fluorescent dye Cy5 (BambL-Cy5). BambL binds with high affinity to fucosylated cell surface receptors and is rapidly taken up together with the bound receptors (ref. 25 and unpublished observation). Initial experiments showed that pressures larger than 100 mbar applied to the basolateral channel occasionally damaged the monolayer above the pore. Therefore, the pressure applied to the basolateral channel was limited to 20 mbar for further experiments. Fig. 3 shows time-lapse images from an experiment in which medium containing BambL-Cy5 was introduced to the basolateral channel and the cells were observed at the level of the chip surface with a confocal microscope. After BambL-Cy5 arrives at the pore (Fig. 3, 10 min), it binds to cells that have access to the pore opening. Importantly, cells distant from the pore do not show BambL-Cy5 signals after the end of the experiment at 180 min. This is even true for BambL-Cy5-stained cells that begin to move away from the pore, which is apparent for the BambL-Cy5-positive cell extending to the left of the pore in Fig. 3 at 140 min that moves further left until 180 min. This indicates that the cell monolayer is attached tightly enough to the chip surface to prevent measurable spreading of BambL-Cy5 within the measurement period of 180 min, even if cells within the monolayer maintain their natural motility.

**Fig. 4:**
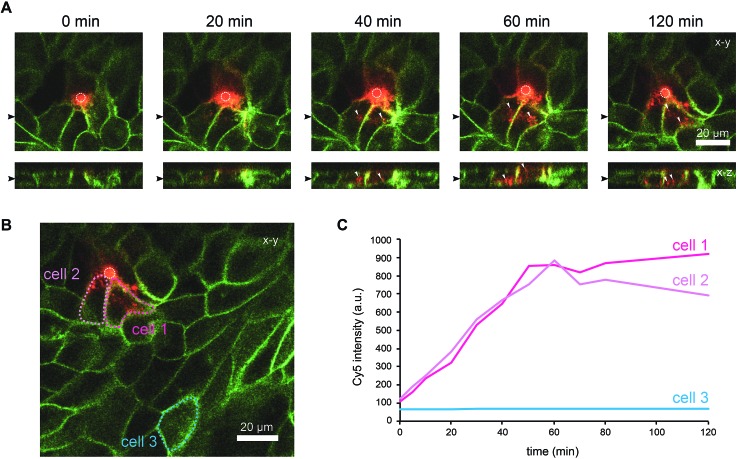
Uptake of ligands by selected cells within an epithelial monolayer. MDCK cells stably expressing ML-GFP (green) were grown as polarised monolayer on the biochip. Medium containing 10 μM BambL-Cy5 (red) was introduced into the basolateral channel and a time-lapse recording with a confocal microscope was started. 0 min indicates the time at which BambL-Cy5 reached the cells through the pore. A white circle indicates the perimeter of the pore. (A) Representative time-lapse images from the recording. For each time point a *x*–*y* section and a *x*–*z* section are depicted, the positions of the respective image sections are indicated by black arrowheads. White arrowheads point to BambL-Cy5 that has been completely taken up by cells. (B) Larger overview image of the same monolayer as in (A), 120 min. The locations of cells contacting the pore (cells 1 and 2) and of a cell further away from the pore (cell 3) are indicated. (C) Graph showing the time course of the BambL-Cy5 signal from the cells indicated in (B).

To further characterise the spreading of BambL-Cy5 after application through the pore, we repeated the experiment and acquired confocal image stacks completely covering the cell monolayer every 10 min (Fig. 4). Again, BambL-Cy5 signals remained restricted to cells that contact the pore (Fig. 4A). Importantly, no staining was apparent at the apical membrane of the cells (Fig. 4A, *x*–*z* sections). This indicates that indeed no paracellular leakage of BambL-Cy5 occurred during the experiment, as was expected from the fact that cells typically formed intact tight junctions above the pore (Fig. 2B). In addition, it can be seen from the *x*–*y* and *x*–*z* sections in Fig. 4A that at later time points BambL-Cy5 was also found in intracellular vesicles that have no contact to the plasma membrane (white arrowheads), which proves that BambL-Cy5 had been successfully taken up by stimulated cells. To determine the kinetics of BambL-Cy5 binding and uptake of the cells, we performed an analysis in which the ML-GFP signal was used to define the outline of cells to measure the time course of the BambL-Cy5 signal from these cells. For comparison, cells at the pore (Fig. 4B, cells 1 and 2) and a control cell further away (Fig. 4B, cell 3) were analysed. As expected, the cell further away from the pore did not show an increase in BambL-Cy5 signal over time (Fig. 4C, cell 3). For the cells contacting the pore, a clear increase of BambL-Cy5 signal occurred over time, which began to saturate after approximately 60 min of stimulation (Fig. 4C, cells 1 and 2). This crucial observation demonstrates that although only cells contacting the pore are affected, sufficient amounts of solutes like BambL-Cy5 can be applied through the pore to saturate the cellular binding and uptake capacity.

Taken together, these experiments show that the biochip enables controlling and changing the basolateral microenvironment of selected cells within an epithelial monolayer. To demonstrate the variability of our technique, we constructed a chip with two pores per channel. As shown in Fig. S4,† this enabled a local stimulation at multiple positions within a polarised monolayer.

### Combined atomic force and fluorescence microscopy

As indicated before, the chip is designed to provide a large opening at the apical side of the cell monolayer in order to enable direct access for the cantilever holder of an AFM. The reason for incorporating this option is that malignant cells often show markedly altered surface topographies and mechanical properties,^20^–^22^ which can be precisely determined by AFM. We would like to add a few considerations for AFM-based characterisation of cells with our chip. Standard PDMS used in microfluidic biochips is approximately 3 orders of magnitude stiffer than epithelial cells (Young's modulus of PDMS ∼ 1 MPa,^27^ Young's modulus of MDCK cells ∼ 0.4 kPa (ref. 28)). When AFM indentation experiments are performed to determine cell elasticity, mainly the mechanical response from a shallow region around the contact area is measured. For example, for typical cell indentation experiments (spherical indenter with 5 μm diameter, indentation depths of 1 μm), it was estimated by finite element analysis that cells are only affected by the indentation to a depth of approximately 4 μm.^29^ Since monolayers of polarised MDCK cells are thicker than 10 μm, the freestanding PDMS membrane, which constitutes the cell substrate in our chip, should not significantly affect AFM elasticity measurements. Furthermore, to avoid transmission of mechanical disturbances to the chip during AFM assays, tubing connected to the chip should be fixed on the stage and microfluidic flow rates or pressures kept constant.

**Fig. 5:**
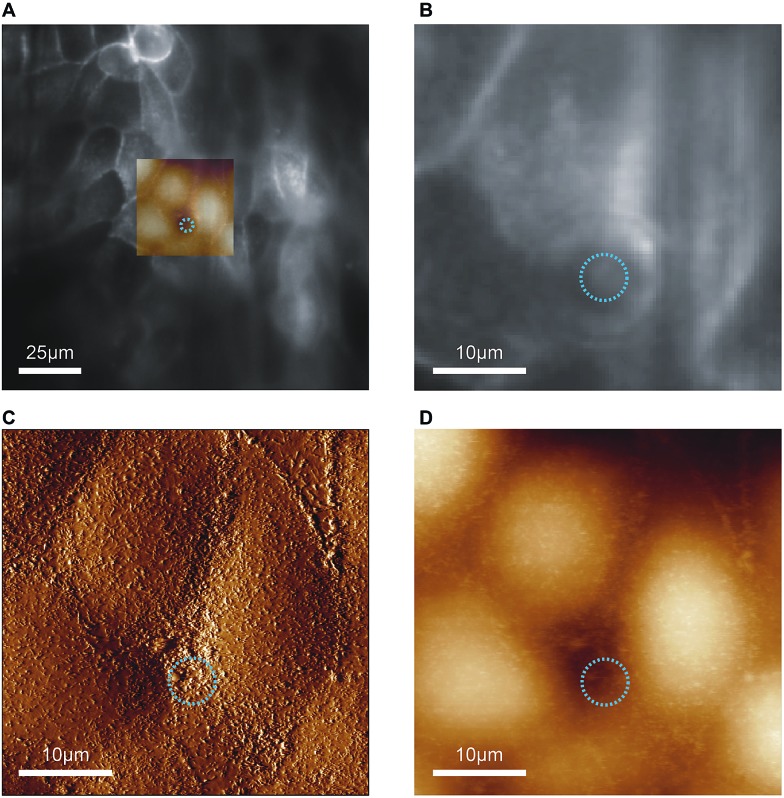
Feasibility of combined atomic force and fluorescence microscopy on the biochip. (A) An overlay of the entire field of view of the fluorescence image with the scanned AFM region is shown. The blue circle indicates the position and diameter of the pore underneath the cell layer. (B) The zoom-in of the optical image shows the distribution of ML-GFP on the cells. (C) Vertical deflection image and (D) height image from contact-mode AFM imaging of the same region as in (B).

In proof-of-concept experiments we showed that combined and correlated AFM and fluorescence microscopy is feasible on the biochip (Fig. 5). In the overlay image (Fig. 5A) fluorescence signals from ML-GFP outlining the cell membranes (Fig. 5B) nicely correlate with the topographical cell perimeters in the contact-mode AFM images (Fig. 5C and D). Especially the AFM vertical deflection image (Fig. 5C) reveals the surface topography at high resolution and fine details such as microvilli can be easily recognised. In addition, cell–cell contacts emerge prominently in Fig. 5C, because they are composed of structures of increased mechanical stability, such as tight junctions, adherens junctions, and an actomyosin ring.^30^

Taken together, the combined AFM and fluorescence microscopy experiments demonstrate that the chip is thin enough to enable high-resolution optical microscopy, but still provides sufficient mechanical stability for AFM-based characterisation.

## Conclusions

All cells sense and respond to their microenvironment. Polarised epithelial cells are surrounded by a specific microenvironment that is determined by neighbouring cells, the extracellular matrix, and soluble molecules, which ensures that individual cells maintain their epithelial phenotype. On the other hand, an altered microenvironment also critically influences processes like malignant transformation and escape of individual cells from the epithelium during epithelial-mesenchymal transformation. However, these initial steps of tumour formation are currently only partially understood.

Here, we present a microfluidic biochip that enables for the first time to actively control the basolateral microenvironment of a confined number of cells within an epithelium. The functional unit of the biochip is a single pore on the basolateral side of the epithelium through which solutes, such as chemokines, cytokines or growth factors, can be precisely applied. Since cells always maintain a small distance of a few ten nm between the substrate and the cell membrane, also pores that are located exactly under a single cell allow a diffusive spreading of soluble stimulants, although slowed down by the extracellular matrix. Therefore, the spreading of stimulants is limited by the density of the extracellular matrix and cellular receptors that immobilise the stimulants. Hence, the concentration and duration of stimulation for a given combination of stimulant and cells should be optimised to prevent excessive diffusive spreading. In addition, as we show exemplarily in Fig. 3, cells within an epithelial monolayer naturally move, which also limits the time a single cell spends directly above the pore.

In proof-of-principle experiments we demonstrated the feasibility of changing the microenvironment of a few adjacent epithelial cells by localised application of the cell-binding lecting BambL. The lectin was subsequently also taken up by epithelial cells contacting the pore, whereas at neighbouring cells that had no pore contact, no BambL was detected. In addition, no paracellular leakage of BambL occurred. Hence, all conditions are fulfilled to adjust and maintain the basolateral microenvironment of few neighbouring cells within an epithelial monolayer.

In future, the described device will enable to study tumour initiation from epithelial cells in a novel way, since it makes it for the first time possible to manipulate the basolateral microenvironment of few epithelial cells within an otherwise undisturbed epithelium. The chip was designed in a way that it allows real-time monitoring of cells with fluorescence microscopy using high-resolution objectives with high NA and low working distances and, in parallel, with AFM. It will allow, for example, the utilisation of fluorescent reporter molecules in live or fixed cells to track changes in the microenvironment or the expression of oncogenes. In addition, long-term cell culture is possible on our chip, thus enabling to directly follow the fate of cells undergoing malignant transformation.

In addition, the localised application of solutes to the basolateral side of epithelia opens up further experimental possibilities, such as to locally probe and image paracellular transport *via* tight junctions.

## Conflicts of interest

There are no conflicts to declare.

## Supplementary Material

Supplementary information
