# Supplementary material for: A microfluidic biochip for locally confined stimulation of cells within an epithelial monolayer
Source: RSC Adv. 2018 Feb 19;8(14):7839–46. doi: 10.1039/c7ra11943g (PMC5830875; doi:10.1039/c7ra11943g)
Supplement: RA-008-C7RA11943G-s001 [file RA-008-C7RA11943G-s001.pdf]

Electronic Supplementary Material (ESI) for RSC Advances.
This journal is © The Royal Society of Chemistry 2018

Supplementary Material

for

A microfluidic biochip for locally confined stimulation of cells within an epithelial monolayer

Roland Thuenauer,[a,b,c,*] Simon Nicklaus,[a,b,c] Marco Frensch,[a,b,c,d] Kevin Troendle,[a,b] Josef Madl,[a,b,c] and Winfried Römer[a,b,c,*]

a. Faculty of Biology, Schänzlestraße 1, Albert-Ludwigs-University Freiburg, 79104 Freiburg, Germany.

b. BIOSS - Centre for Biological Signalling Studies, Schänzlestraβe 18, Albert-Ludwigs-University Freiburg, 79104 Freiburg, Germany.

c. Freiburg Center for Interactive Materials and Bioinspired Technology (FIT), Georges-Köhler-Allee 105, Albert-Ludwigs-University Freiburg, 79110 Freiburg, Germany

d. International Max Planck Research School for Molecular and Cellular Biology, Max Planck Institute of Immunobiology and Epigenetics, Stübeweg 51, 79108 Freiburg, Germany

* to whom correspondence should be addressed: roland.thuenauer@gmail.com and winfried.roemer@bioss.uni-freiburg.de

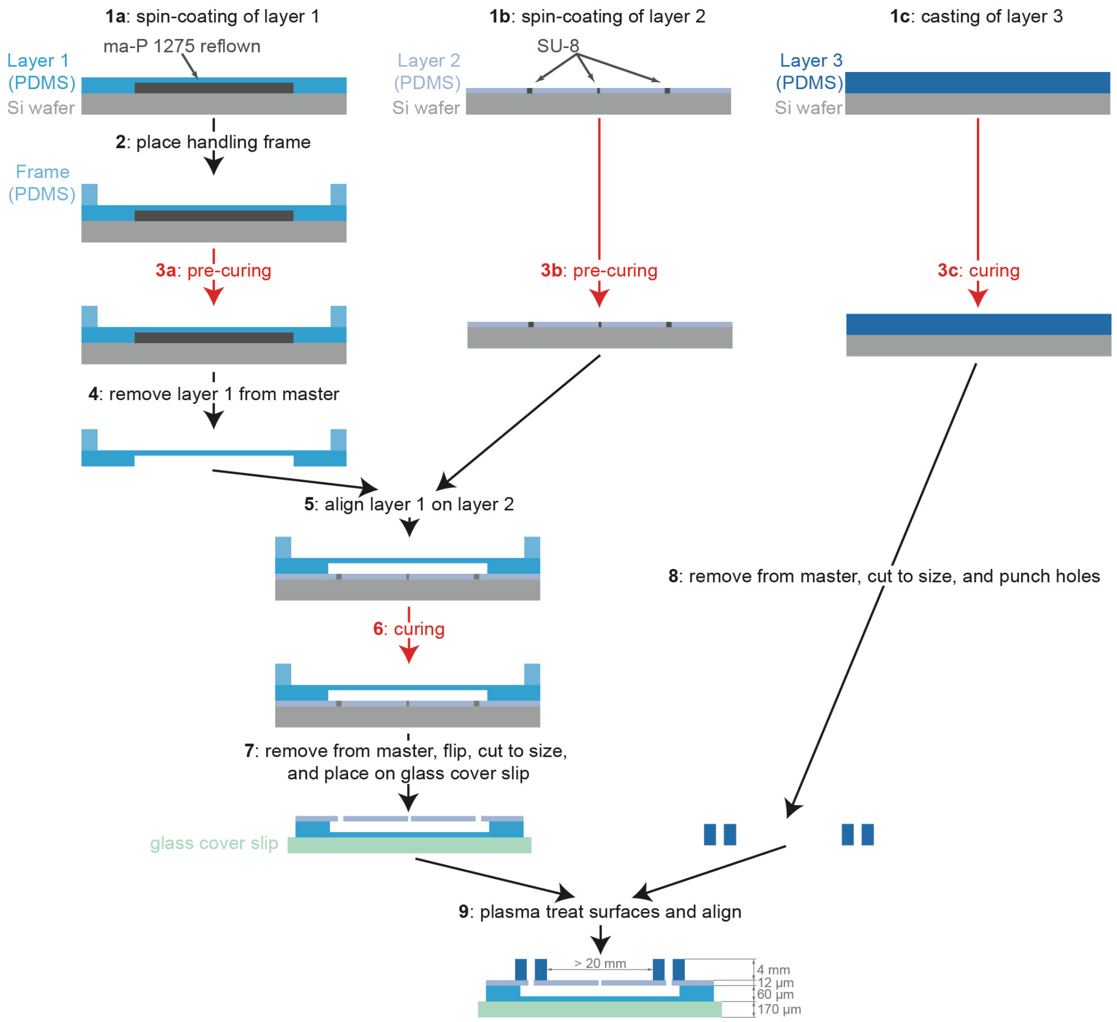

Fig. S1: Outline of the manufacturing procedure for the biochip.
PDMS structures are shown in different shades of blue. Masters are shown in different shades of grey and consist of silicon wafers and structures made from the photoresists SU-8 and ma-P 1275. The glass cover slip is shown in light green.

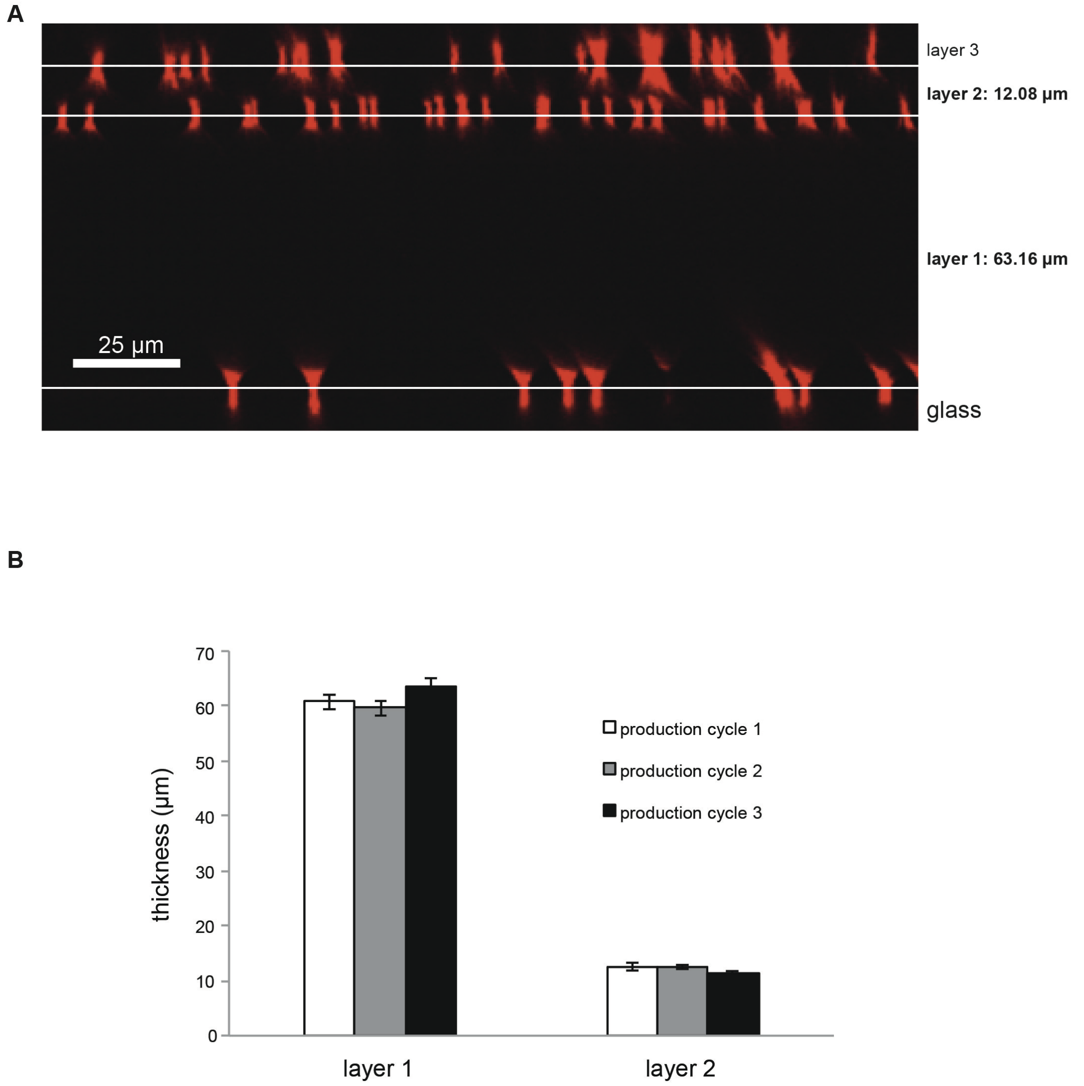

Fig. S2: Determination of the thicknesses of layers 1 and 2.
(A) During production of the chip, fluorescent TetraSpeck beads were incorporated between each layer. This allowed determining the thicknesses of layers 1 and 2 from the image stack recorded by a confocal microscope. A representative x-z section is shown and white lines indicate the layer interfaces. (B) Results of the thickness determination as described in (A). For each production cycle 5 chips were analysed, error bars represent standard error mean (SEM).

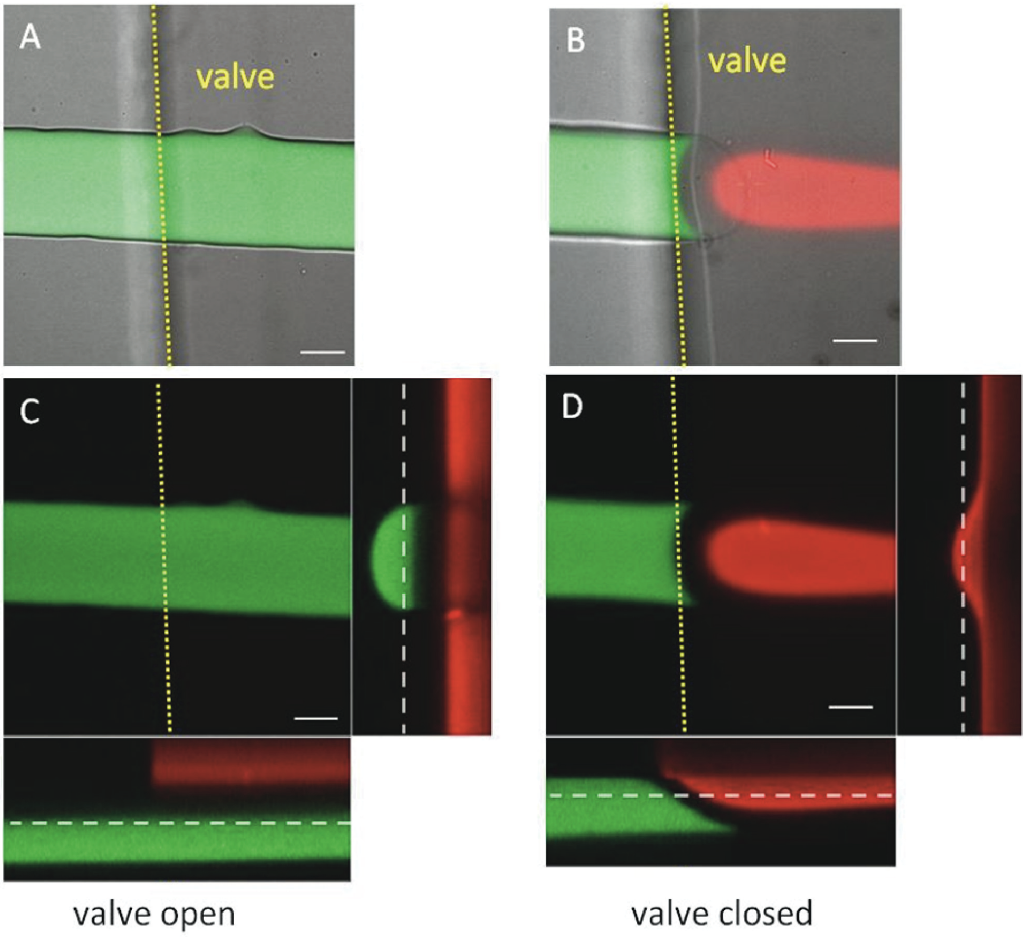

Fig. S3: Verification of valve functionality.
The flow channel was filled with green fluorescent solution and the actuator channel was filled with red fluorescent solution. In (A) and (C) the valve is open, in (B) and (D) the valve is closed. (A) and (B) show x-y sections of overlays of a white light transmission image and fluorescence images. (C) and (D) show x-y sections (middle panels), x-z sections (lower panels), and y-z sections (right panels) of the fluorescence images. Yellow dashed lines indicate the edge of the actuator chamber of the valve. White dashed lines indicate the positions of the x-y sections in the x-z and y-z section images. The scale bars correspond to 20 µm.

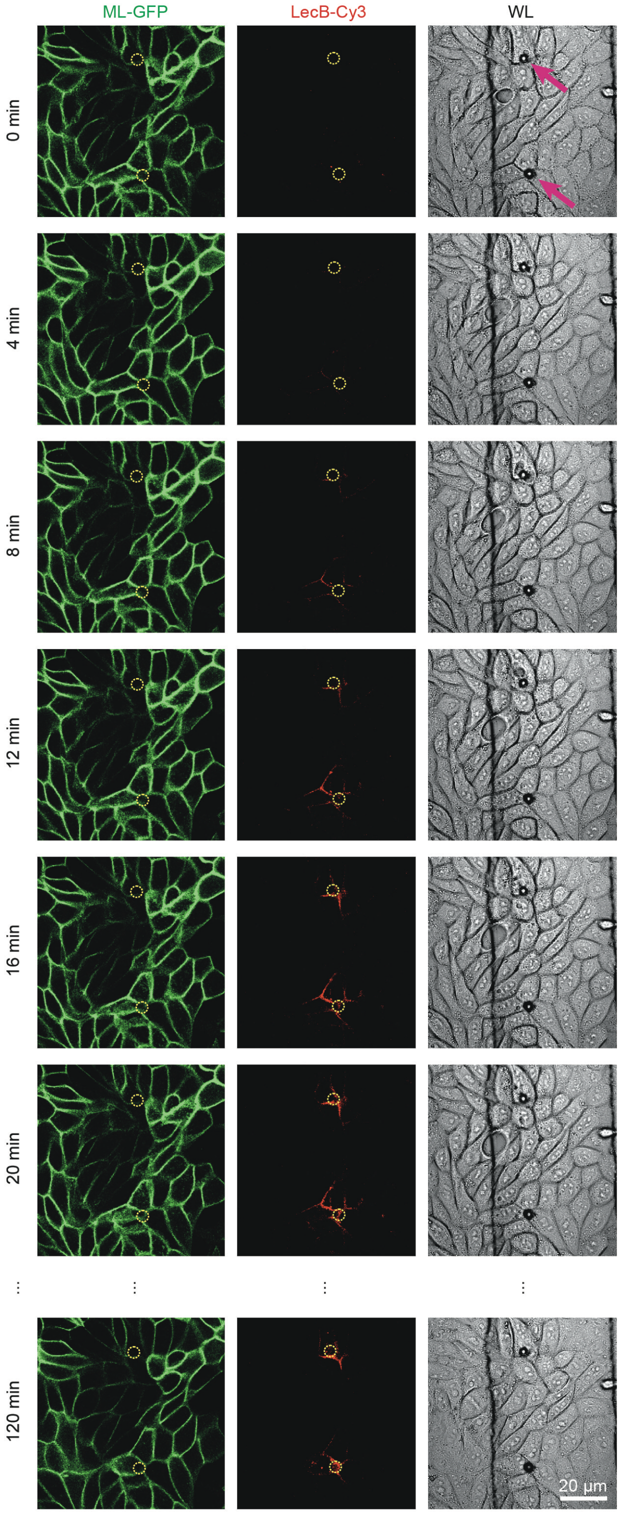

Fig. S4: Local stimulation with the *Pseudomonas aeruginosa* lectin LecB at multiple positions in a chip with two pores per channel.

A chip was produced with two pores per channel. In the top right white light (WL) transmission image the two magenta arrows point to the positions of the pores. In the fluorescence images yellow circles outline the pores. For the experiment MDCK cells expressing ML-GFP (green) were grown as confluent monolayer on the chip and 5 µM of LecB tagged with Cy3 (LecB-Cy3; red) was introduced to the basolateral channel at 2 min while an image plane on which the cells are attached to the chip was observed with a confocal microscope.
